# Anisotropic terahertz transmission induced by the external magnetic field in La_0.67_Ca_0.33_MnO_3_ film

**DOI:** 10.1063/4.0000123

**Published:** 2021-10-05

**Authors:** Hongying Mei, Peng Zhang, Shile Zhang, Ruxian Yao, Haizi Yao, Feng Chen, Zhenyou Wang, Fuhai Su

**Affiliations:** 1 Henan Key Laboratory of Smart Lighting, School of Information Engineering, Huanghuai University, Zhumadian 463000, China; 2 Fuyang Normal University, Fuyang 236037, China; 3 High Magnetic Field Laboratory, HFIPS, Chinese Academy of Sciences, Hefei 230031, China; 4 Great Bay Area Research Institute, Aerospace Information Research Institute, Chinese Academy of Science, Guangzhou 510530, China; 5 Key Laboratory of Materials Physics, Institute of Solid State Physics, HFIPS, Chinese Academy of Sciences, Hefei 230031, China

## Abstract

A systemic investigation of the terahertz (THz) transmission of La_0.67_Ca_0.33_MnO_3_ film on the (001)-oriented NdGaO_3_ substrate under external magnetic field and low temperature have been performed. The significant THz absorption difference between the out-of-plane and the in-plane magnetic field direction is observed, which is consistent with the electrical transport measurement using the standard four-probe technique. Furthermore, we find that the complex THz conductivities can be reproduced in terms of the Drude Smith equation as the magnetic field is perpendicular to the film plane, whereas it deviates from this model when the in-plane magnetic field is applied. We suggest that such anisotropies in THz transport dynamics have close correspondences with the phase separation and anisotropic magnetoresistance effects in the perovskite-structured manganites. Our work demonstrates that the THz time-domain spectroscopy (TDS) can be an effective non-contact method for studying the magneto-transport properties of the perovskite-structured manganites.

## I. INTRODUCTION

The physical properties of the perovskite-structured manganites at the low temperature and high external magnetic field condition have attracted intensive attention due to their interesting crystal structures and strong interplays among charge, lattice, spin, and orbital degrees of freedom. This kind of material system exhibits plenty of novel physics phenomena, including the colossal magnetoresistance (CMR) effect,^1^ anisotropic magneto-resistance (AMR),^2^ metal–insulator transition (MIT),^3^ and phase separation (PS).^4^ The extensive investigations in the last decades suggest that this type of material is sensitive to the external conditions, such as temperature, magnetic field, substrate strain, and chemical doping.^5–10^ Among them, the AMR effect is very important and has broad applications in magnetic recording technologies like magnetic read heads and sensors of magnetic materials.^11^ The AMR in manganites depends on the amplitude of the applied magnetic field and the angle between the direction of the magnetic field and the film plane.^12^ Consequently, it can be classified into two types, i.e., the in-plane AMR and the out-of-plane AMR, which stems from the spin–orbit coupling effect and the phase competition, respectively. A close relationship exists between the AMR effect and the PS in the perovskite manganites,^13,14^ and both can be modulated by the external magnetic field. The PS is characterized by the competition between the ferromagnetic metallic (FMM) phase and the antiferromagnetic insulating (AFI) phase in perovskite-structured manganites.^15^ The transition, ranging from the AFI-dominant PS to the FMM-dominant PS in manganites, also named the melting transition under the high magnetic field condition, which were usually explored with microscopy techniques.^16,17^ However, this electrical method is not applicable for the phase at nanometer scale due to the extremely strict measurement condition of the larger melting magnetic field for manganites.^18–21^ Although the electrical transport resistivity measurements can characterize the PS and AMR, it is inconvenient because they all have to make contacts to the sample.^15^ Thus, a new method in the noncontact and nondestructive fashion is highly desired to probe the transport dynamics of the manganites.

As an optical method, the terahertz time-domain spectroscopy (THz-TDS) is a powerful tool to explore the charge-carrier dynamics for perovskite-structured manganites in the far-infrared spectral region.^11,22,23^ The THz-TDS allows for the direct determination of the complex conductivity without the Kramers–Kronig transformation. Moreover, it can also be used for characterizing the charge carrier dynamics on the nanometer scale.^22^ For example, Lloyd–Hughes *et al.* discovered the nanometer scaled colossal terahertz magnetoresistance in the vertically aligned nanocomposites of La_0.7_Sr_0.3_MnO_3_ and found that the transport mechanisms are different from the direct current colossal magnetoresistance.^24^ To date, the THz-TDS is rarely employed to explore the PS and the AMR effects, especially in the perovskite-structured manganites.

In this work, we utilize the THz-TDS to study the AMR and the PS effects in the La_0.67_Ca_0.33_MnO_3_ film. We find that the THz transmission strongly depends on the magnetic field direction with respect to the film plane, which are consistent with the direct electrical transport measurement using the standard four-probe technique in the Physical Property Measurement System (PPMS, Quantum Design). In addition, the obtained THz complex conductivities in the frequency domain are analyzed using the Drude–Smith model, revealing the carrier scattering mechanism across the phase transition under the magnetic field. Our work demonstrates that the THz-TDS is an excellent technique to study the physical properties of the manganites under extreme conditions, which might also be applicable to other similar materials.

## II. METHODS

A 40 nm thick epitaxial La_0.67_Ca_0.33_MnO_3_ film (in the *xy*-plane) was grown on an orthorhombic 0.5 mm thick NdGaO_3_ (001) substrate using the pulsed laser deposition method reported in the previous work.^14^ The substrate temperature and O_2_ pressure were set at 735 °C and 45 Pa, and the laser pulse energy and repetition rate were kept at 2 J/cm^2^ and 5 Hz, respectively.

**FIG. 1.**
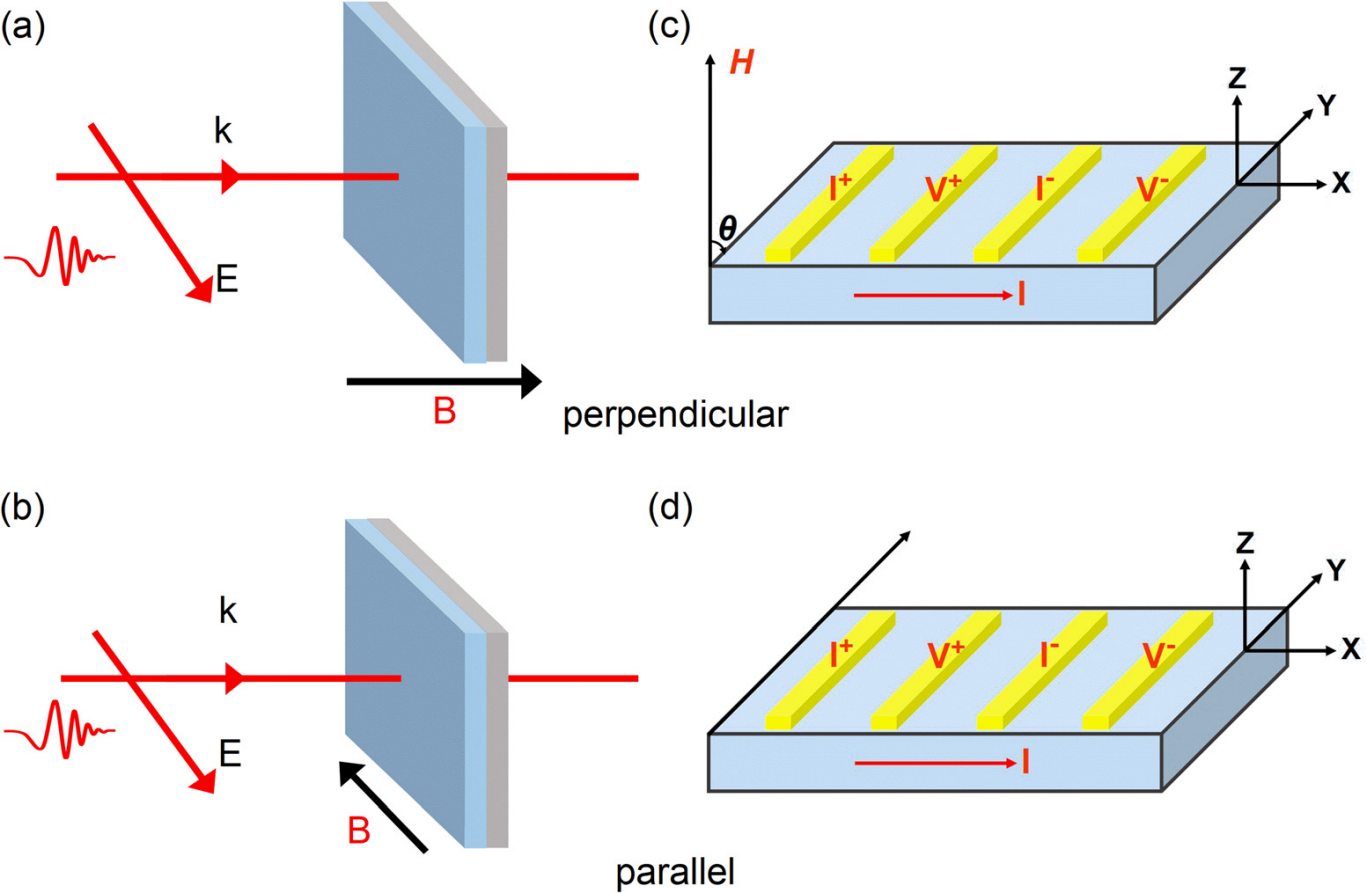
Schematic illustrations for measuring the AMR at the low-temperature and high magnetic field with the THz-TDS method (a), (b), and the electrical transport measurement (c), (d). The magnetic field is perpendicular to the film plane in (a) and (c), and the magnetic field is parallel to the film plane in (b) and (d).

For measuring the THz transmission spectra under external magnetic field, a home-built THz magneto-optical time-domain spectroscopy (MO-TDS) system was applied.^25^ The THz-TDS was carried out in a standard transmission configuration with a mode-locked Ti:sapphire laser system. The duration, center wavelength, and the repetition rate of the laser pulses are 150 fs, 800 nm, and 76 MHz, respectively. The broadband THz pulses were generated from a low temperature-GaAs photoconductive antenna. The free-space electro-optic sampling with a ZnTe single crystal is employed to detect the THz waveforms in the time domain. A magneto-optical superconducting magnet system (Oxford Spectromag SM4000) is used to tune the magnetic field and temperature conditions for the sample property measurement. The sample is fixed on a copper cold-finger with a 2 mm-diameter hole. The experiments were performed in a dry nitrogen environment to avoid the water vapor absorption interference. In the THz-TDS measurement, the film plane was set as either perpendicular (out of plane) or parallel (in plane) to the external magnetic field, while the p-polarized THz electric field $\vec{E}$ is kept parallel to the film plane as shown in Figs. 1(a) and 1(b). To vary the temperature, the zero-field cooling procedure was taken, that is, the sample was cooled down to the target temperature from room temperature at zero magnetic field before taking magnetic-field dependent measurements for each round of experiment. The THz transmission spectra at the magnetic field ranging from 0 to 4 T and the temperature of 210, 105, and 30 K were measured, respectively.

The magneto-transport property was obtained by a physical property measurement system (PPMS) with assembling a superconducting magnet. In the PPMS measurement, the film plane was also rotated to be perpendicular (out of plane) or parallel (in plane) with the external magnetic field direction, the schematic diagram is depicted in Figs. 1(c) and 1(d). The measured temperature and the magnetic field conditions are same as those in the THz transmission measurement, respectively.

## III. RESULTS AND DISCUSSION

**FIG. 2.**
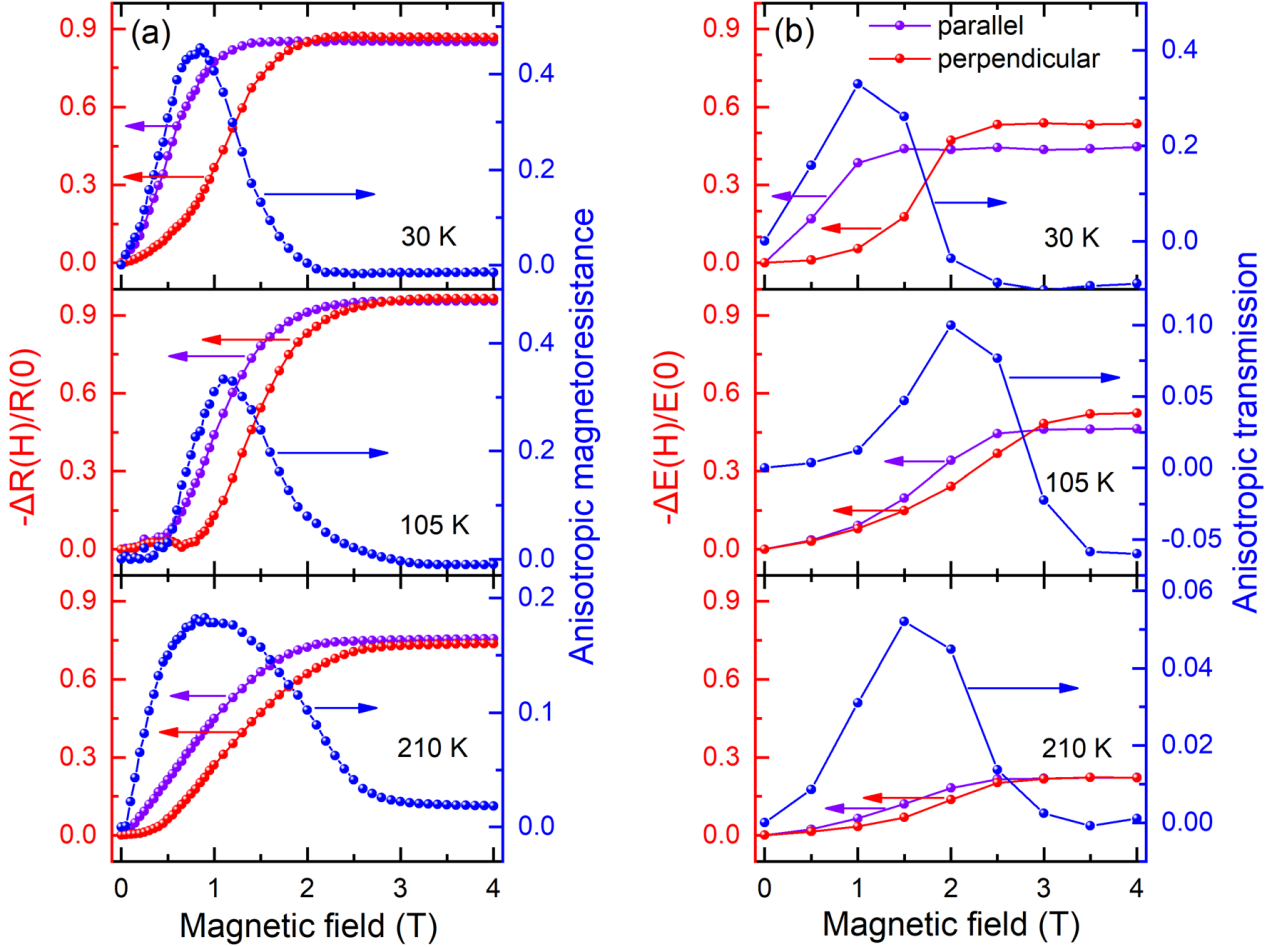
The relative resistance (a) (red and purple dotted lines) and the THz relative transmission (b) (red and purple dotted lines) of the La_0.67_Ca_0.33_MnO_3_ film measured by the THz-TDS and electrical transport measurement at temperatures of 30, 105, and 210 K, respectively. The anisotropic magnetoresistance (a) (blue dotted lines) and anisotropic transmission (b) (blue dotted lines) are also shown. The errors are smaller than the size of the symbols of experimental data.

Figure 2(a) presents the differential resistance −ΔR(H)/R(0) of the La_0.67_Ca_0.33_MnO_3_ film at different magnetic fields and temperatures. −ΔR(H)/R(0) drastically increases at the beginning and finally saturates with increasing magnetic field. Such a phenomenon corresponds to the PS effect induced by the phase transition from the FMM-dominant phase to the AFI-dominant phase, which is consistent with the Ref. 14. The melting field H_*M*_ of the original FMM phase, which is determined from the intersection point of the tangents before and after the transition field, is 1.5 and 2.5 T at 30 K, 2.5 and 3 T at 105 K, and 2.5 and 3 T at 210 K for the magnetic field parallel and perpendicular to the film plane, respectively. The apparent discrepancies between these two curves at each temperature imply that there is an out-of-plane AMR effect in the La_0.67_Ca_0.33_MnO_3_ film.^14^ Interestingly, the H_*M*_ of the former condition is all higher than that of the latter condition, implying that the z-axis, which is perpendicular to the film plane, is the hard axis. The AMR shows a nonmonotonic variation with H, and at H > H_*M*_, the film enters a uniform FMM state at which the AMR effect becomes negligible. The amplitude of the AMR in the PS region is about 45%, 33%, and 18% at 30, 105, and 210 K, respectively. At high temperatures, the AFM order plays a dominant role, and the spin arrangement is almost isotropous. After the low temperature transition to the FMM-dominant state, the spin direction arranges orderly, and the MR in this direction has a certain increase. Thus, the amplitude of the AMR gradually enhances with decreasing temperature.

In the next, we turn to discuss the magnetic-field dependences of the THz transmission of the La_0.67_Ca_0.33_MnO_3_ film. The THz amplitude transmission can be expressed in terms of the film conductivity *σ*(*H*), under magnetic field *H*, that is,^26,27^

$$T=\frac{E_{\mathrm{substrate}+\mathrm{film}}}{E_{\mathrm{substrate}}}=\frac{1+n}{1+n+Z_{0}\sigma (H)d},$$ (1)

here, *n* is the refractive index of the sample, Z_0_ = 377 Ω is the impedance of the free space, and *d* is the film thickness. Therefore, the differential transmission with respect to the zero-field value, ΔT/T_0_ = [T(H) − T(0)]/T(0), can be derived from Eq. (1),

$$\frac{\Delta T}{T_{0}}=-\frac{Z_{0}d\Delta \sigma }{1+n+Z_{0}d\sigma_{0}}.$$ (2)

Here, Δσ denotes the differential conductivity between with [ σ(H)] and without [ σ(0)] the external magnetic field, i.e., Δσ = σ(H)−σ(0). Therefore, it is clear that the ΔT/T_0_ is proportional to the magnetic-field induced change in THz conductivity, i.e., ΔT/ $T_{0}∼\Delta \sigma$. Figure 2(b) shows the obtained ΔT/T_0_ according to the THz pulse peak transmission of the La_0.67_Ca_0.33_MnO_3_ film at different magnetic fields and temperatures. One can see that the −ΔE(H)/E(0) almost shares the same magnetic-field behavior with the resistance results, exhibiting obvious PS effect and strong out-of-plane AMR effect. The amplitude of the anisotropic transmission is about 33%, 10%, and 5% at 30, 105, and 210 K, respectively, and increases with the decreasing temperature. The consistent behavior between the THz transmission and the resistance measurements implies that the THz-TDS is an effective method to identify the PS and AMR effects in the La_0.67_Ca_0.33_MnO_3_ film. However, it is worth noting that the amplitude values are a little different from the resistance results. Such a phenomenon should result from the optical conductivity being sensitive to the excitation wavelength, and the wavelength range in our measurement is relatively narrow (0–3 THz) compared to the total conductivity.

**FIG. 3.**
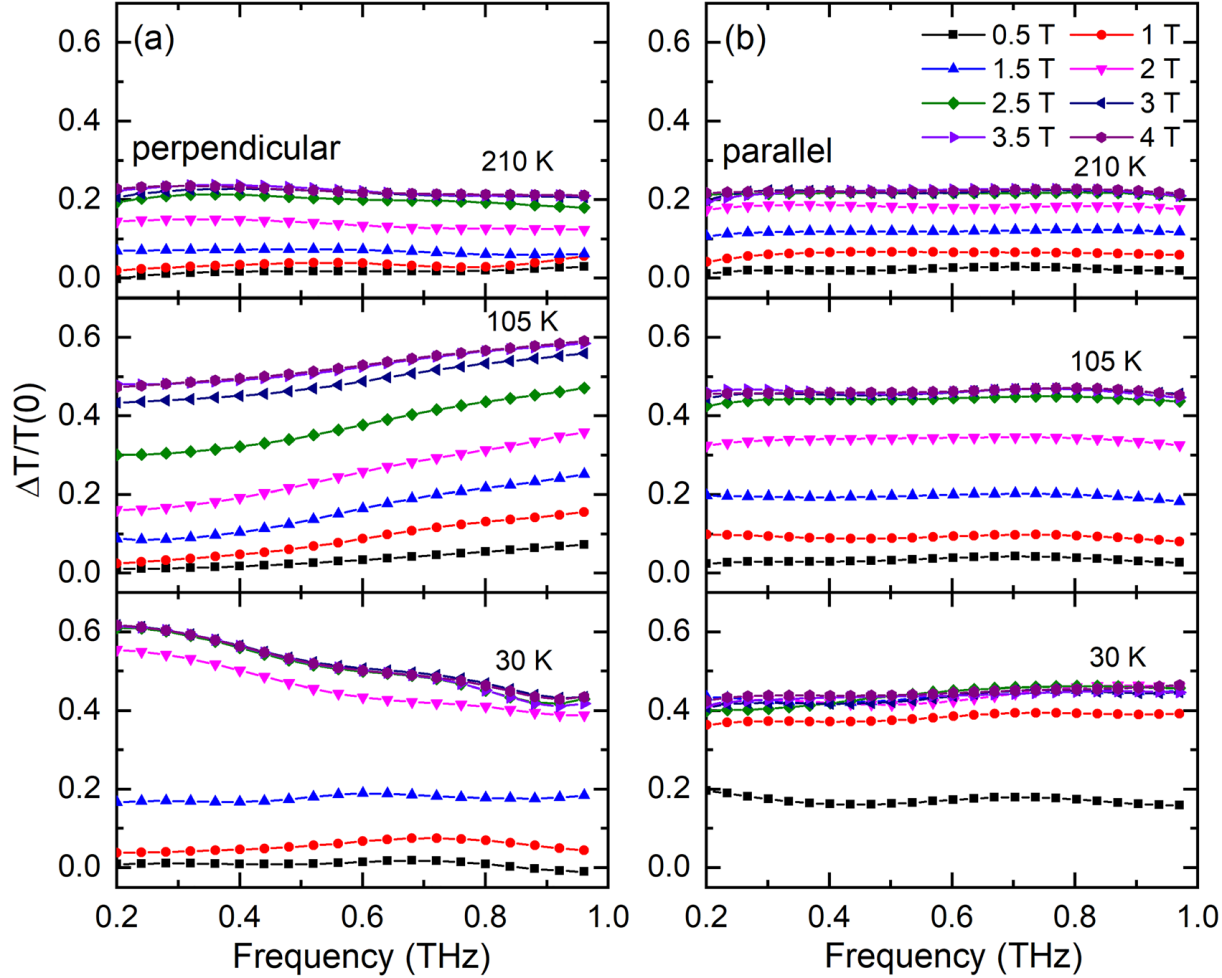
THz relative transmissivity vs the frequency at various magnetic field strengths, directions, and temperatures. (a) The magnetic field is perpendicular to the film plane. (b) The magnetic field is parallel to the film plane. The errors are smaller than the size of the symbols of experimental data.

The frequency dependence of the THz relative transmissivity at different magnetic fields is also analyzed in detail to study the correlation between the AMR and PS effects. As shown in Fig. 3, the transmissivity at different conditions (temperature and orientation) all increases with the increasing magnetic field and then becomes constant, which is in line with the resistance results. It indicates that the THz transmissivity is also susceptible to the PS effect. In addition, the relative transmissivity at 210 K almost shows the same tendencies with the frequency for these two magnetic field configurations, whereas they become clearly different at 105 and 30 K, i.e., the transmissivity increases at 105 K and decreases at 30 K for the configuration that the magnetic field is perpendicular to the film plane with the increasing frequency, the transmissivity keeps constant in the measured frequency range for the other configuration. Such results should result from the AMR effect in the La_0.67_Ca_0.33_MnO_3_ film.

**FIG. 4.**
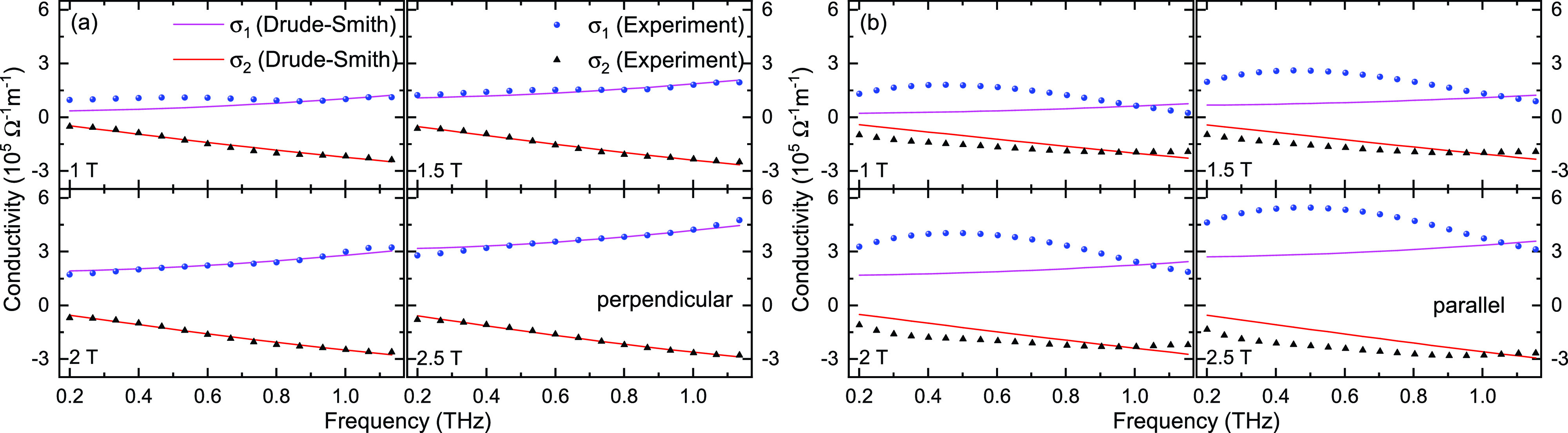
The complex conductivity with the magnetic field perpendicular to the film plane (a) and the magnetic field parallel to the film plane (b) fitted with the Drude–Smith model for the La_0.67_Ca_0.33_MnO_3_ film at 105 K and the magnetic field ranges from 1 T to 2.5 T. The blue solid circles (experiment) and the corresponding lines (Drude–Smith fitting) are the real part $\sigma_{1}$ of the THz conductivity, and the black solid triangles (experiment) and the corresponding lines (Drude–Smith fitting) are the imaginary part $\sigma_{2}$ of the THz conductivity. The errors are smaller than the size of the symbols of experimental data.

Furthermore, we proceed to examine the conductivity spectra of the La_0.67_Ca_0.33_MnO_3_ film in the THz region. Combining with the real and imaginary parts of the E_*substrate*+*film*_(*ω*, B) and E_*substrate*_(*ω*), the complex conductivity [*σ*(*ω*) = $\sigma_{1}$(*ω*) + i $\sigma_{2}$(*ω*)] of the film can be determined by the formula:^26,27^

$$\frac{E_{\mathrm{substrate}+\mathrm{film}}(\omega ,B)}{E_{\mathrm{substrate}}(\omega )}=\frac{1+n}{1+n+Z_{0}\sigma (\omega ,B)d},$$ (3)

where *n* = 5 is the refractive index of the substrate NdGaO_3_. Due to the high effective mass m^*^ = 23m_0_ for the La_0.67_Ca_0.33_MnO_3_ film,^23^ we neglect the cyclotron resonance frequency $\omega_{c}$ = eB/m^*^. To understand the basic features of the complex conductivities, we employ the Drude–Smith model to analyze the experimental data,^28,29^

$$\sigma (\omega )=\frac{\sigma_{0}}{1-i\omega \tau }\left[1+\frac{c_{1}}{1-i\omega \tau }\right],$$ (4)

where the *dc* conductivity $\sigma_{0}$ = ${\text{ne}}^{2}\tau$/m^*^, n is the carrier density, m^*^ is the carrier effective mass, *τ* is the relaxation time, and c_1_ denotes the fraction of the initial velocity of an electron after scattering events and varies between −1 and 0, which corresponds to localization and Drude formula, respectively. The complex conductivities and the fitted results at 105 K are shown in Fig. 4. For the configuration that the magnetic field is perpendicular to the film plane, the real conductivities $\sigma_{1}$ increases and the imaginary part $\sigma_{2}$ decreases as the frequency is increased, all the parts fit very well with the Drude–Smith model. However, for the parallel configuration, the real conductivity $\sigma_{1}$ and the imaginary part $\sigma_{2}$ all decline as the frequency is increased. These typical behaviors are incompatible with the Drude–Smith model. It should be mentioned that the Drude–Smith model is based on the assumption that the carrier backscattering persists for only single scattering event and has some limitations for the inhomogeneous media.^30,31^ Jiang *et al.* observed the formation of striped magnetic domains in La_0.325_Pr_0.3_Ca_0.375_MnO_3_ films by magnetic force microscopic (MFM) imaging, depending on the in-plane strains and direction of external magnetic field.^14^ Such orientated magnetic domains were proposed to have close correspondences with the observed colossal anisotropic resistivity. Herein, we speculate that the external magnetic field can give rise to the similar magnetic domains, containing the ferromagnetic-metal inclusions, in our studied La_0.67_Ca_0.33_MnO_3_. For the parallel configuration, these inhomogeneous striped magnetic domains, aligned with magnetic field direction, likely lead to more anisotropic in-plane scattering events, such as skew scattering, quantum tunneling, which make the THz conductivities spectra deviating from Drude–Smith model. In contrast, the in-plane anisotropic effect of magnetic domains distribution should be small in the perpendicular configuration. In future, more elaborate methods, such as effective medium theories (EMTs), Monte Carlo simulation, or calculations based on quantum effects, need to be developed to exactly understand the transport mechanism in the THz frequency region.

**FIG. 5.**
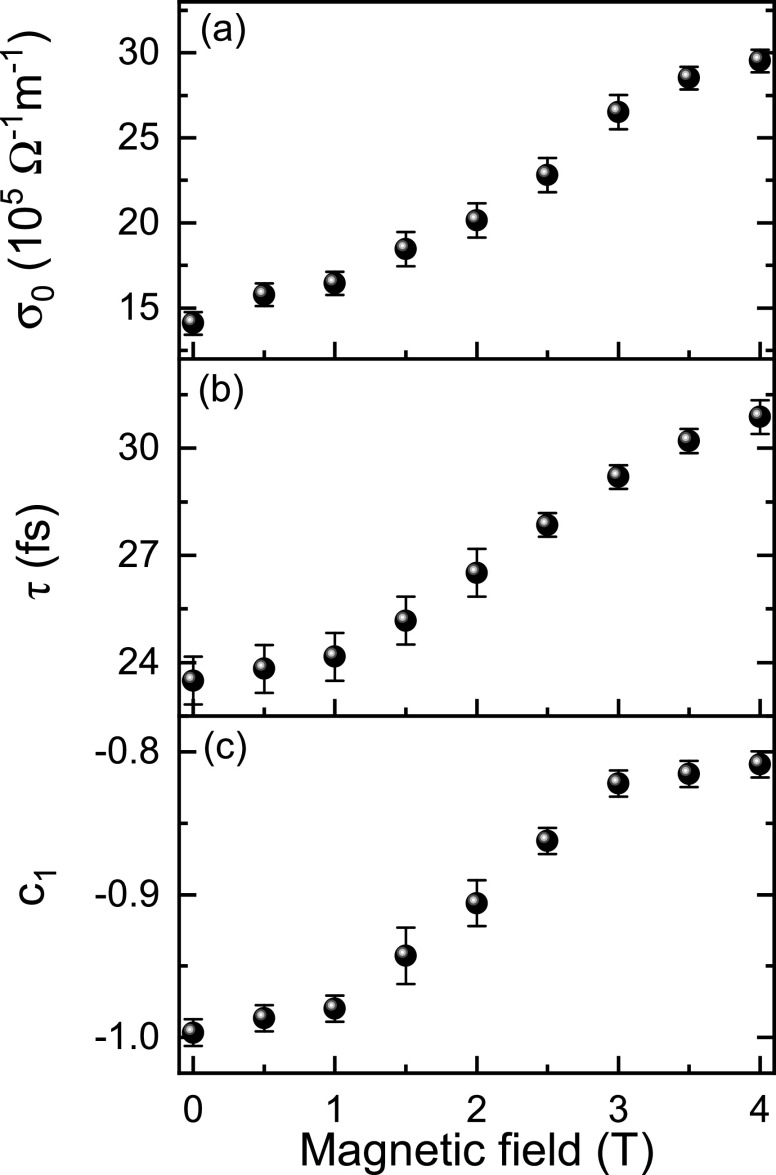
The *dc* THz conductivity $\sigma_{0}$ (a), relaxation time *τ* (b), and persistence of velocity c_1_ (c) of the La_0.67_Ca_0.33_MnO_3_ film with the magnetic field perpendicular to the film plane at 105 K.

Figure 5 presents the fitted *dc* conductivity ( $\sigma_{0}$), relaxation time (*τ*), and persistence of the carrier velocity (c_1_) using the Drude–Smith model. The *dc* conductivity ( $\sigma_{0}$) gradually increases with the increasing magnetic field, as well as the relaxation time *τ*, implying that the magnetic field promotes the movement of the conductive electrons. It is also one of the important conditions to enhance the *dc* conductivity of this material. As can be seen, the factor c_1_, characterizing the localization of conducting electrons, increases with magnetic field and becomes moderate at high magnetic fields. With the increasing magnetic field, the antiferromagnetic insulating state is gradually suppressed, and the magnetic domains, composed with ferromagnetic metal inclusions, start to form and become larger with the magnetic field.^14^ Therefore, the magnetic field dependence of c_1_ factor is consistent with the evolution of the ferromagnetic metal phase.

## IV. CONCLUSION

In conclusion, the magnetic field dependent THz transmission is investigated for the La_0.67_Ca_0.33_MnO_3_ film that is deposited on a (001) oriented NdGaO_3_ substrate by THz-TDS. The results showed similar behaviors to that of the electrical resistance measurement, such as the AMR and the PS effects. More information on the THz conductivity is extracted and checked with the Drude–Smith model. We found that it does not fit the conductivity for the condition when the magnetic field is parallel to the film plane, but works well when the magnetic field is perpendicular to the film plane. The factor for persistence of carrier velocity (c_*j*_), the *dc* conductivity $\sigma_{0}$, and the electronic relaxation time *τ* of the La_0.67_Ca_0.33_MnO_3_ film are, thus, able to be obtained with the Drude–Smith model. In conclusion, our work suggests that the THz-TDS is applicable to detect the AMR and PS effects of the La_0.67_Ca_0.33_MnO_3_ film without any physical contacts. Since it is less susceptible to the shape and size of the sample as well, it could be a new way to study the AMR and PS effects in the perovskite-structured manganites.

## Data Availability

The data that support the findings of this study are available from the corresponding authors upon reasonable request.
